# Comparison of Workplace Social Capital between the Pre- and Post-Restriction Eras of Physician Working Hours: A Nationwide Repeated Cross-Sectional Study

**DOI:** 10.31662/jmaj.2025-0444

**Published:** 2026-02-20

**Authors:** Hirohisa Fujikawa, Takuya Aoki, Masato Eto

**Affiliations:** 1 Department of General Medicine, Juntendo University Faculty of Medicine, Bunkyo-ku, Tokyo, Japan; 2 Center for General Medicine Education, School of Medicine, Keio University, Shinjuku-ku, Tokyo, Japan; 3 Department of Medical Education Studies, International Research Center for Medical Education, Graduate School of Medicine, The University of Tokyo, Bunkyo-ku, Tokyo, Japan; 4 Division of Clinical Epidemiology, The Jikei University School of Medicine, Minato-ku, Tokyo, Japan; 5 Section of Clinical Epidemiology, Department of Community Medicine, Graduate School of Medicine, Kyoto University, Sakyo-ku, Kyoto, Japan

**Keywords:** workplace social capital, vertical trust, horizontal trust, working hour restriction, resident

## Abstract

**Introduction:**

Workplace social capital (WSC), defined as the features of social organization that promote coordination and cooperation for mutual benefit, is a relevant construct that contributes positively to employee and organizational wellness, and has recently attracted wide attention. However, factors associated with WSC in the field of medical education have not been investigated, including regarding the effect of physician working hour restrictions. Thus, the aim of the study was to investigate differences in the WSC of resident physicians before and after the April 2024 introduction of restrictions on physician working hours in Japan.

**Methods:**

We conducted a nationwide repeated cross-sectional survey in 25 hospitals across Japan. Pre- and post-restriction data were obtained in July and August 2022 and in December 2024, respectively. We evaluated WSC using the Japanese medical resident version of the WSC Scale, which comprises horizontal trust (i.e., trust in co-workers) and vertical trust (i.e., trust in supervisors) subscales. We used the total score and its domain scores, all of which range from 1 to 5, with higher scores indicating greater WSC, as outcome variables. We created a dummy variable (1 = post-restriction data [December 2024], 0 = pre-restriction data [July-August 2022]) and used it as the explanatory variable. We used a linear mixed-effects model to adjust clustering within hospitals and individual covariates.

**Results:**

We analyzed data for 428 respondents (pre-restriction, 246; post-restriction, 182 participants; man, 272). After adjusting possible confounders and clustering within hospitals, physician working hour restrictions were significantly associated with greater vertical trust (adjusted mean difference, 0.17; 95% confidence interval, 0.02 to 0.32). No clear trend was observed in the association among restrictions, total WSC score (adjusted mean difference, 0.13; 95% confidence interval, −0.01 to 0.26), and horizontal trust score (adjusted mean difference, 0.10; 95% confidence interval, −0.05 to 0.24).

**Conclusions:**

This nationwide multicenter study revealed significant vertical trust score differences between pre- and post-physician working hour restrictions. The implementation of physician working hour restrictions has improved vertical trust. These findings contribute to the literature on potential benefits of working hour restrictions on the organization of physician life and enhancement of patient care quality.

## Introduction

Physician working hour restrictions originated in the Western countries but have since been adopted in Asia and other global regions as a structural response to longstanding concerns about excessive workloads and fatigue among physicians, particularly those in the early stages of training ^(1)^. The Accreditation Council for Graduate Medical Education first introduced the restrictions in 2003 in the U.S., and they have been revised several times since then ^(2)^. The European Working Time Directive became law in 1998, limiting working hours to protect the health and safety of workers in all occupations in the European Union ^(2)^. In the 2010s, Korea and Taiwan introduced duty hour restrictions for physicians ^(3), (4)^. Prolonged working hours for physicians have also long been an issue in Japan, and social concern over the issue has grown ^(5)^. As a result, working hour restrictions for physicians were introduced in April 2024 throughout Japan ^(6)^. These limit physicians to 960 hours of overtime per year ^(7), (8), (9)^. Physicians in special circumstances, such as medical residents and physicians at the stage of training when they must develop advanced clinical skills, in addition to physicians working at hospitals providing community and emergency care, are permitted to work up to 1,860 hours of overtime per year ^(7), (8), (9)^.

Although these restrictions are primarily intended to protect physician health and ensure patient safety, they may also influence the social and organizational climate of clinical training environments ^(1), (10), (11)^. Reduced fatigue, increased perceptions of fairness, and improved work-life balance can reshape how physicians perceive their relationships with colleagues and supervisors. These shifts can be understood through the concept of workplace social capital (WSC). It is defined as a workplace resource that is concerned with the perceptions of employees regarding trust, reciprocity, and network interactions that exist both among peers and among individuals across different hierarchical levels or organizations ^(12)^. WSC may prove beneficial to organizational life because it allows employees to engage in collective behavior and provides access to further resources ^(13)^. Indeed, studies have indicated that there are positive links between WSC and job satisfaction, psychological well-being, and work engagement ^(14), (15), (16)^, and negative links between WSC and emotional exhaustion, job stress, sub-optimal health, and mortality ^(17), (18), (19), (20)^. Moreover, a growing body of literature in health care settings has shown that greater WSC is associated with better patient care quality ^(21), (22)^. Thus, WSC is crucial for people of working age and health care professionals.

However, little is known about ways system level policies such as working hour restrictions affect WSC among physicians in clinical training environments. In fields other than medical education, several studies have explored some determinants of WSC. A Chinese study indicated that cultural factors, such as Chinese Confucian values, had mixed effects on WSC. Respect for authority and altruism were positively associated with WSC, whereas acceptance of authority was found to be negatively linked with WSC ^(23)^. Workplace size was identified as an organizational determinant, with larger units associated with lower WSC ^(24)^. Psychological work environments were positively associated with WSC ^(23)^. In addition, employee demographics also play a role, with a higher proportion of manual and male workers associated with reduced WSC, whereas increased proportion of temporary employees were associated with greater WSC ^(24)^. Working hour restrictions have the potential to improve WSC overall because they may signal institutional support and reinforce perceptions of fairness.

Thus, the aim of this study was to evaluate the differences in perceived WSC among medical trainees between pre- and post-implementation of the working hour restrictions in Japan in April 2024, which provided a natural experiment for observing the organizational effects of this policy. We anticipated that the findings would provide international medical educators with insights into WSC, which could lead to a better organizational life for physicians and enhanced patient care quality.

## Methods

### Study design, setting, and participants

To examine differences in WSC between the pre- and post-restriction of physician working hours, we decided to obtain new post-restriction data in this study in December 2024, and to use data from a previous survey conducted from July to August 2022 as pre-restriction data ^(15), (25)^. We proceeded in the following three steps. First, we created an online questionnaire on SurveyMonkey (www.surveymonkey.com). Second, we contacted the training directors of 32 postgraduate clinical training hospitals in Japan that participated in the WSC study from July to August 2022 and asked for their cooperation with our study. Twenty-five of the 32 hospitals agreed to participate in the post-restriction survey. Third, we emailed the resident physicians of these hospitals through their training directors with web link to the questionnaire. In the email, the residents were asked to read a research description document, which informed them of the anonymous and voluntary nature of the study, and to complete the online, anonymous, self-administered questionnaire. We sent two follow-up email reminders during the survey period. As an incentive, respondents in the post-restriction survey entered a draw for one of ten ¥5,000 gift cards.

### Measures

#### Outcome variable: WSC

We used the Japanese medical resident version of the Workplace Social Capital (JMR-WSC) Scale to evaluate WSC ^(25)^. The original English WSC Scale, developed by Kouvonen et al. ^(26)^, was translated and cross-culturally adapted to the Japanese postgraduate medical education context, which led to the development of the JMR-WSC Scale in 2023 ^(25), (26), (27)^. The scale has eight items, each of which is rated on a five-point Likert scale ranging from 1 = strongly disagree to 5 = strongly agree. The score of the scale is the average of all eight items and ranges from 1 to 5, such that higher scores indicate a higher level of WSC. The scale also comprises two subscales that measure horizontal trust (Q1-5) and vertical trust (Q6-8). Horizontal trust is defined as the relations of trust and reciprocity between individuals and groups occupying the same hierarchical level ^(28), (29), (30)^, whereas vertical trust is defined as norms of respect and networks of trusting relations among people who interact across explicit, formal, or institutionalized gradients of power and authority ^(29), (30), (31), (32)^. Accordingly, we also calculated the average for each subscale, ranging from 1 to 5, with higher scores indicating a higher level of each dimension of WSC ^(25)^.

#### Explanatory variable: Pre- or post-implementation of physician working hour restriction

A dummy variable was used to distinguish between data before and after the implementation of physician working hour restrictions. The dummy variable had a value of 1 for data obtained in December 2024, which corresponded to the period after the implementation of the restrictions, and a value of 0 for data from the prior WSC study, which was conducted from July to August 2022. We used this dummy variable as an explanatory variable.

#### Covariates

In this study, covariates included gender, postgraduate years (junior or senior resident), clinical department, hospital type (community or university hospital), and hospital size (≤500 beds, 501-800 beds, 801-1,000 beds, or ≥1,001 beds). We classified clinical departments into the following three groups based on previous literature: internal medicine (internal medicine, pediatrics, and general medicine), surgical medicine (general surgery, neurosurgery, obstetrics and gynecology, ophthalmology, orthopedics, otorhinolaryngology, plastic surgery, and urology), or other departments (anesthesiology, dermatology, emergency medicine, psychiatry, and radiology) ^(15), (33), (34)^. Variables probably lying on the causal pathway (e.g., actual working hours, perceived social support, and work engagement) were not included in the model to avoid post-treatment bias.

### Statistical analysis

First, descriptive statistics were conducted to analyze the collected data, with continuous data reported as means and standard deviations and categorical data as frequencies and percentages.

Second, to evaluate WSC score differences between pre- and post-implementation of physician working hour restrictions, we used a linear mixed-effects model (random intercept model). The model includes random effects for hospitals and both individual-level covariates (gender, postgraduate years, clinical department) and hospital-level covariates (hospital type and hospital size) as fixed effects. Because this primary analysis suggested that residents in the post-working hour restriction era exhibited significantly greater vertical trust than did those in the pre-working hour restriction era (as will later be described in detail), subsequent exploratory subgroup analysis was conducted to ascertain whether the observed directional pattern (i.e., post > pre) was consistent across various hospital types and sizes. We also conducted subgroup analyses for the total WSC score and horizontal trust score. We chose complete case analysis owing to the small amount of missing data. A two-tailed p-value <0.05 was considered significant. All statistical analyses were performed with SPSS version 29.0 (IBM Corp).

### Ethical considerations

We conducted this study in accordance with the Declaration of Helsinki and relevant guidelines. Before participation, all study participants provided informed consent by checking a consent box. This study was approved by the ethical committee of Keio University School of Medicine (number 20231224).

## Results

**Figure 1.**
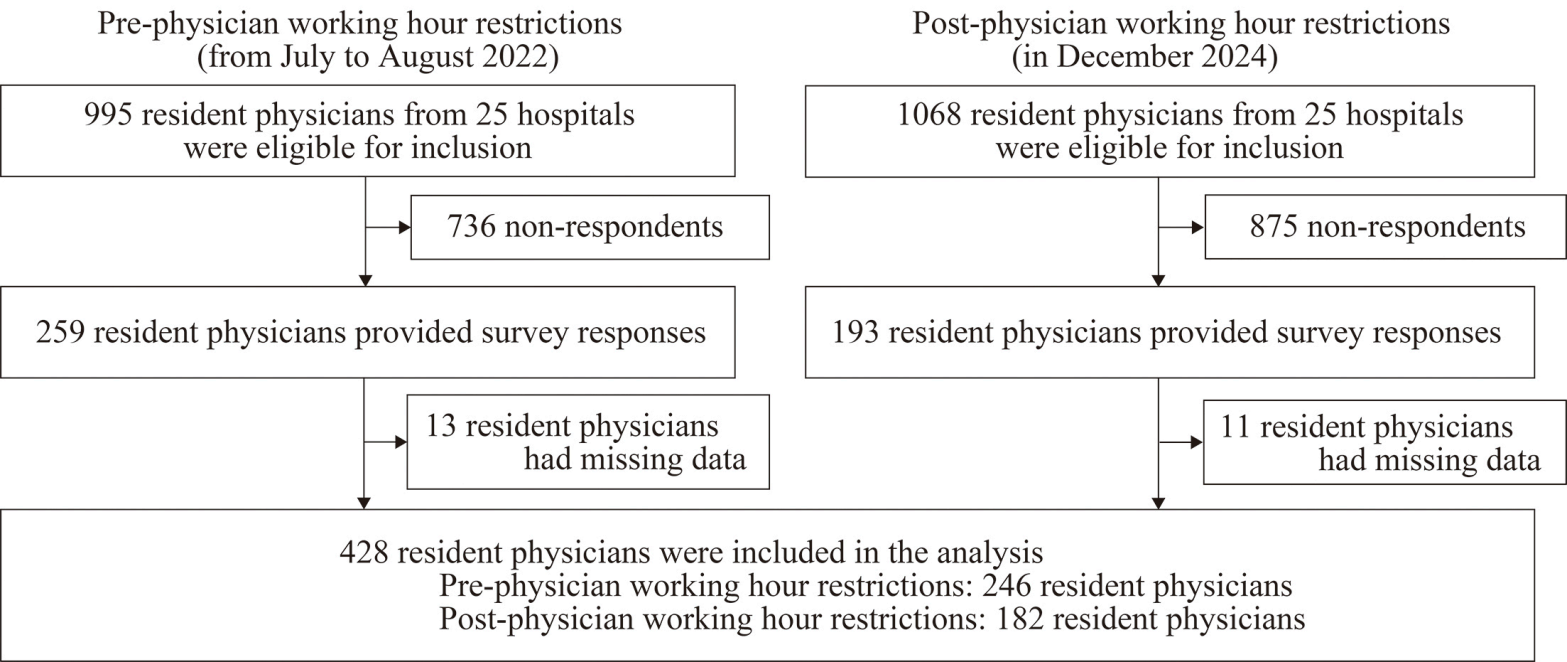
Participant flowchart. A total of 32 postgraduate clinical training hospitals participated in the pre-restriction survey conducted in 2022. For the post-restriction phase, the training directors of all 32 hospitals were contacted and invited to participate in the follow-up survey. Of these, 25 hospitals consented to participate. Accordingly, resident physicians from these 25 hospitals were included in both the 2022 and 2024 survey waves.

Table 1 lists the characteristics of the 25 participating hospitals. Figure 1 shows the participant flowchart. Of 2,063 eligible participants, 452 completed our survey. After excluding 24 participants with missing data, we included the remaining 428 participants (response rate, 20.7%; pre-working hour restriction, 246 participants; post-working hour restriction, 182 participants) in our final analysis. Table 2 summarizes the characteristics of the participants. Most participants were men (272, 63.6%), had a postgraduate year of 1-2 (274, 64.0%), belonged to a community hospital (252, 58.9%), belonged to a hospital with 1,001 or more beds (158, 36.9%), and belonged to the internal medicine department (227, 53.0%).

**Table 1.** Characteristics of the Participating Hospitals.

Characteristics	n (%)
Hospital type	
Community hospital	23 (92)
University hospital	2 (8)
Hospital size	
≤500 beds	15 (60)
501-800 beds	6 (24)
801-1,000 beds	3 (12)
≥1,001 beds	1 (4)
Hospital location	
Hokkaido and Tohoku	4 (16)
Kanto	4 (16)
Chubu	5 (20)
Kinki	3 (12)
Chugoku and Shikoku	3 (12)
Kyushu	6 (24)

**Table 2.** Participant Characteristics.

Characteristics	Total (N = 428)	Pre-working hour restriction^a^ (n = 246)	Post-working hour restriction^b^ (n = 182)
Gender, n (%)			
Woman	154 (36.0)	90 (36.6)	64 (35.2)
Man	272 (63.6)	155 (63.0)	117 (64.3)
Non-binary	2 (0.5)	1 (0.4)	1 (0.5)
PGY, n (%)			
1-2 (junior residents)	274 (64.0)	155 (63.0)	119 (65.4)
3-6 (senior residents)	154 (36.0)	91 (37.0)	63 (34.6)
Hospital type, n (%)			
Community hospital	252 (58.9)	134 (54.5)	118 (64.8)
University hospital	176 (41.1)	112 (45.5)	64 (35.2)
Hospital size, n (%)			
≤500 beds	137 (32.0)	73 (29.7)	64 (35.2)
501-800 beds	96 (22.4)	53 (21.5)	43 (23.6)
801-1,000 beds	37 (8.6)	17 (6.9)	20 (11.0)
≥1,001 beds	158 (36.9)	103 (41.9)	55 (30.2)
Clinical department, n (%)			
Internal medicine^c^	227 (53.0)	142 (57.7)	85 (46.7)
Surgical medicine^d^	119 (27.8)	65 (26.4)	54 (29.7)
Other departments^e^	82 (19.2)	39 (15.9)	43 (23.6)
WSC scale,^f^ mean (SD)			
Total	4.11 (0.71)	4.06 (0.74)	4.18 (0.66)
Horizontal trust	4.01 (0.77)	3.97 (0.78)	4.06 (0.74)
Vertical trust	4.28 (0.80)	4.21 (0.84)	4.37 (0.74)

PGY: postgraduate year; SD: standard deviation; WSC: workplace social capital. ^a^Data obtained from July to August 2022. ^b^Data obtained in December 2024. ^c^Internal medicine, pediatrics, and general medicine. ^d^General surgery, neurosurgery, obstetrics and gynecology, ophthalmology, orthopedics, otorhinolaryngology, plastic surgery, and urology. ^e^Anesthesiology, dermatology, emergency medicine, psychiatry, and radiology. ^f^Ranging from 1 to 5, with higher scores indicating greater WSC.

Table 3 lists the adjusted mean differences in JMR-WSC Scale scores between pre- and post-restrictions of physician working hours. There was no clear trend in association between the physician working hour restriction and the JMR-WSC Scale total scores after adjustment for possible confounders and clustering within hospitals (adjusted mean difference, 0.13; 95% confidence interval [CI], −0.01 to 0.26). Among the WSC subscales, resident physicians in the post-restriction era had significantly greater vertical trust than did those in the pre-restriction era, after adjusting for possible confounders and clustering within hospitals (adjusted mean difference, 0.17; 95% CI, 0.02 to 0.32). Conversely, there were no significant score differences in horizontal trust in the workplace between pre- and post-restrictions of working hours (adjusted mean difference, 0.10; 95% CI, −0.05 to 0.24). Supplementary Table 1 presents the results of the additional subgroup analysis. Across all strata, the point estimates for post-physician working hour restrictions were all positive, indicating directional consistency (i.e., residents in the post-working hour restriction era perceived greater WSC than did those in the pre-working hour restriction era). For vertical trust, statistical significance, as defined by 95% CI that excludes the null value, was observed among community hospitals and hospitals with 500 or fewer beds. In the remaining subgroups, the point estimates remained positive, yet the 95% CI crossed zero.

**Table 3.** Associations between Physician Working Hour Restrictions and WSC Scores^a^ (N = 428).

Outcome^b^	Unadjusted mean difference (95% CI)	Adjusted^c^ mean difference (95% CI)
WSC total score	0.12 (−0.02 to 0.25)	0.13 (−0.01 to 0.26)
WSC subscale scores		
Horizontal trust	0.09 (−0.06 to 0.23)	0.10 (−0.05 to 0.24)
Vertical trust	0.16 (0.01 to 0.31)^*^	0.17 (0.02 to 0.32)^*^

CI: confidence interval; WSC: workplace social capital. ^a^Random intercept model; reference group: pre-working hour restrictions ^b^All scores range from 1 to 5, with higher scores indicating greater WSC ^c^Adjusted for gender, postgraduate years, clinical department, hospital type, and hospital size * p < 0.05.

## Discussion

In this study, we investigated the differences in WSC between pre- and post-implementation of physician working hour restrictions. The results indicated that resident physicians in the post-working hour restriction era exhibited significantly greater vertical trust scores on the JMR-WSC Scale than did those in the pre-working hour restriction era. In contrast, there were no significant score differences in total WSC and horizontal trust in the workplace between pre- and post-restrictions of physician working hours. These findings will be insightful for international medical educators and policymakers.

A large body of literature has assessed the influence of physician working hour restrictions in terms of patient safety, physician education, and physician well-being. Conversely, few studies have evaluated its impact on workplace dynamics. A Canadian qualitative study found that duty hour restrictions led to decreased physical and mental exhaustion, and ultimately enhanced teamwork ^(35)^. However, to the best of our knowledge, no quantitative study has been performed to evaluate whether working hour restrictions lead to changes in workplace dynamics. The present study is of considerable significance because it quantitatively tested the hypotheses generated by the previously mentioned Canadian qualitative research and reinforced the evidence for the impact of working hour restrictions on workplace dynamics.

Our multilevel analysis revealed that medical residents in the post-working hour restriction era exhibited significantly greater vertical trust than did those in the pre-working hour restriction era. Additional subgroup analysis showed that the post-pre working hour restrictions difference was evident across diverse settings, although it achieved statistical significance only in specific strata. Although differences in subgroup sample sizes may contribute to varying estimate precision, this factor alone is unlikely to fully account for the observed pattern. It is also possible that the magnitude of change differed across institutional contexts (e.g., hospital size and/or type), such that working hour restrictions were sufficient to shift vertical trust in some settings but not others. In light of this possibility, we interpret the subgroup findings as suggestive of contextual heterogeneity rather than as a simple power artifact. Although the subgroup analyses indicated possible contextual variation, the overall analysis still revealed a significant improvement in vertical trust after the implementation of physician working hour restrictions. This overall change in vertical trust may be explained by several underlying mechanisms. First, implementation required open communication and transparent decision-making regarding workstyle between supervisors and resident physicians ^(36)^. Through this process, residents may have observed that their concerns were being addressed (e.g., fatigue, overwork), which may have engendered trust in supervisors. Second, nationwide working hour restrictions policies explicitly strengthened supervision standards and accountability of supervising physicians ^(37)^, requiring training programs to define levels of supervision and ensure that an identifiable supervisor is ultimately responsible for each patient. Such clearly defined, policy-backed supervisory structures could improve residents’ perceptions of reliability and availability of supervisors. Third, the working hour policies required training programs to monitor working hours and adjust schedules when necessary to alleviate excessive workload demands ^(37)^. These procedural safeguards signal fair process and responsiveness by leadership, which in turn support trust in those who allocate work and set schedules. Thus, these policy-linked, supervisor-facing changes would offer a coherent explanation for the selective improvement in vertical trust.

In contrast to the increase in vertical trust, we saw no significant differences in horizontal trust in the workplace between the periods before and after the implementation of physician working hour restrictions. Although it is possible that reduced working hours could increase off-duty opportunities for social connection among peers, contextual factors may attenuate such gains in our setting. For example, in Japan’s early residency training system, residents frequently rotate through multiple departments in brief periods ^(38), (39)^, limiting the sustained, repeated co-working with the same peers that appears crucial for fostering peer-to-peer trust. However, this rotational pattern could possibly affect relationships with supervisors in different ways, indicating that working hour restrictions may not influence all types of workplace trust in the same direction. Against this backdrop, our null finding for horizontal trust should be interpreted with caution: it remains unclear whether the absence of a significant difference reflects a genuine lack of improvement or simply insufficient statistical power given our relatively small sample size. Further investigation to ascertain whether restrictions would lead to better horizontal trust in a larger sample size is warranted. Moreover, additional studies are required to explore the mechanism through which the restrictions and the horizontal component of WSC may or may not be associated.

Although the observed mean difference of 0.17 points (Cohen’s d ≈ 0.2) may appear small according to conventional statistical benchmarks, it may represent a potentially policy-relevant signal. WSC represents a collective attribute―encompassing trust, reciprocity, and shared norms among colleagues―that typically evolves gradually and resists rapid change. Even small numerical increases in WSC may therefore reflect tangible improvements in the psychosocial work environment. A large Finnish cohort study showed that each one-point increase in WSC was associated with a 19% reduction in all-cause mortality risk ^(19)^. Although we do not infer clinical or educational thresholds from that study, it illustrates how modest differences in WSC can translate into potentially meaningful long-term benefits. However, the field lacks established evidence regarding the interpretability of the WSC Scale, and few studies have quantified the magnitude of score change required to achieve tangible improvements in clinical or educational outcomes. Future research should therefore link changes in WSC to concrete outcomes (e.g., reduced burnout or turnover intention) to provide clearer benchmarks for interpreting score differences.

To our knowledge, this is the first study to evaluate differences in resident physicians’ WSC before and after the implementation of restrictions on physician working hours. The nationwide introduction of these restrictions provided the crucial opportunity for a natural experiment to evaluate our research question. The data were based on a nationwide multicentered survey across Japan. In addition, the instrument used to measure WSC in the study (i.e., WSC Scale) has been shown to be both reliable and valid and has been widely used internationally ^(25), (26)^. Accordingly, our findings were significant, with relatively high external validity. Although this study was conducted in a single country in Asia, its findings will provide in-depth insights for medical educators and policymakers internationally, given that physician working hour restriction has been widely adopted in many countries ^(2), (6), (7)^ and that a considerable body of evidence has suggested that WSC has benefits (e.g., better well-being, greater work engagement, greater job satisfaction, lower exhaustion, and better job quality) ^(14), (15), (16), (17), (18), (19), (20), (21), (22)^.

The subgroup findings suggest that the implications of working hour restrictions for WSC may differ substantially between smaller/community hospitals and larger/university hospitals. In our analyses, improvements were detectable in smaller/community hospitals, whereas no significant change was observed in larger/university hospitals. This divergence is broadly consistent with insights from organizational psychology and occupational health: larger/university hospitals tend to have more hierarchical structures and complex vertical management dynamics ^(40), (41)^. In such settings, improvements in WSC may depend more on internal organizational reforms than solely on working hour restrictions. Conversely, smaller/community hospitals often have flatter structures, shorter communication lines, and more consistent supervisor-trainee contact, which may allow working hour restrictions to translate more directly into enhanced perceptions of support and fairness. In this sense, at least in the context of working hour restrictions, smaller/community hospitals may function as more supportive and accessible organizations for physicians.

This study was subject to several potential limitations. First, it was conducted under a cross-sectional design and therefore precludes any determination of causality. Second, the response rate was relatively low. It is likely that resident physicians with lower WSC would not provide responses to our questionnaire. In addition, given that the participating hospitals participated in our survey voluntarily, they may be representative of hospitals with a greater interest in medical education and/or occupational health. Thus, caution should be exercised when interpreting and generalizing the findings of this study. Third, there is a possibility of misclassification regarding clinical departments due to the self-reported nature of the data. In particular, the categorization of “general medicine” was based on participants’ own interpretation of the response label. Because institutional terminology (e.g., general medicine, general internal medicine, family medicine, hospitalist unit) varies across hospitals in Japan ^(42), (43)^, some degree of inconsistency in how respondents classified their department cannot be ruled out. However, we focused on broader groupings (internal medicine-related vs surgery-related vs other) in our linear mixed-effects model; we expect any such misclassification to be nondifferential and unlikely to substantially bias the results. Fourth, because the number of individuals within each subgroup was modest, the subgroup findings should be interpreted with caution. Larger studies with more balanced subgroup sizes are warranted to clarify whether the effects differ by institutional characteristics. Fifth, as described earlier, although this study showed statistically significant score differences in the vertical component of the WSC, the clinical and educational relevance of the differences remains unclear. Future research should evaluate the interpretability of the WSC Scale, which might confirm the significance of this study.

### Conclusions

This nationwide, multicentered study revealed an association between the physician working hour restrictions and vertical trust in the workplace. The introduction of physician working hours apparently led to better vertical trust. These findings offer novel insights into the impact of physician work-hour restrictions from the perspective of workplace dynamics, a perspective that differs from considerations of patient safety, and physician education and well-being. Improved WSC has the potential to improve the organizational life of physicians and enhance patient care quality.

## Article Information

### Acknowledgments

The authors thank all study participants. The authors also thank ChatGPT-5 from OpenAI for its valuable assistance in refining the academic writing.

### Author Contributions

Hirohisa Fujikawa conceived the study with input from Takuya Aoki and Masato Eto. Hirohisa Fujikawa conducted the data analysis with support from Takuya Aoki. Hirohisa Fujikawa drafted the manuscript. Finally, all authors discussed, proofread, and approved the final version of the manuscript.

### Conflicts of Interest

None

### Ethical Approval Statement

This study was conducted in accordance with the Declaration of Helsinki and relevant guidelines. This study was approved by the ethical committee of Keio University School of Medicine (number 20231224). All study participants provided written informed consent by ticking the consent box in the web questionnaire.

## Supplement

Supplementary Material
