# Supplementary material for: Comparison of Workplace Social Capital between the Pre- and Post-Restriction Eras of Physician Working Hours: A Nationwide Repeated Cross-Sectional Study
Source: JMA J. 2026 Feb 20;9(2):502–10. doi: 10.31662/jmaj.2025-0444 (PMC13058744; doi:10.31662/jmaj.2025-0444)
Supplement: Supplementary Material [file 2433-3298-9-2_0502-s001.pdf]

**Supplementary Table 1. The Results of the Subgroup Analyses Elucidating WSC Score Differences between Pre- and Post-implementation of Physician Working Hour Restrictions.**[a]

| **WSC total score** | | |
|---|---|---|
| **Subgroup** | **Adjusted mean difference** | **95% CI** |
| Community hospital | 0.17 | 0.01 to 0.33* |
| University hospital | 0.09 | −0.15 to 0.32 |
| Hospital with ≤500 beds | 0.13 | −0.07 to 0.33 |
| Hospital with > 500 beds | 0.12 | −0.06 to 0.29 |
| **Horizontal trust score** | | |
| **Subgroup** | **Adjusted mean difference** | **95% CI** |
| Community hospital | 0.11 | −0.06 to 0.28 |
| University hospital | 0.11 | −0.15 to 0.36 |
| Hospital with ≤500 beds | 0.05 | −0.18 to 0.27 |
| Hospital with > 500 beds | 0.12 | −0.06 to 0.30 |
| **Vertical trust score** | | |
| **Subgroup** | **Adjusted mean difference** | **95% CI** |
| Community hospital | 0.25 | 0.06 to 0.43* |
| University hospital | 0.06 | −0.20 to 0.31 |
| Hospital with ≤500 beds | 0.26 | 0.04 to 0.47* |
| Hospital with > 500 beds | 0.12 | −0.08 to 0.32 |

Abbreviation: CI, confidence interval; WSC, workplace social capital

[a] Random intercept model, adjusted for individual-level covariates (gender, postgraduate years, clinical department) and hospital-level covariates (hospital type and hospital size); Reference group: pre-working hour restrictions

* $p < 0.05$
